# Improving Care for Childhood Obesity: A Quality Improvement Initiative

**DOI:** 10.1097/pq9.0000000000000412

**Published:** 2021-05-19

**Authors:** Komal F. Satti, Susanne E. Tanski, Yike Jiang, Auden McClure

**Affiliations:** From the * Dartmouth Hitchcock Medical Center, Lebanon, N.H.; † Baylor College of Medicine, Texas Children’s Hospital, Houston, Tex.

## Abstract

Obesity affected 13.7 million children in the United States in 2015. The American Academy of Pediatrics (AAP) offers an evidence-based approach to obesity management, but adherence to recommendations is suboptimal. Our objective was to improve provider adherence to the AAP recommendations for care of patients with obesity by making systematic changes in our practice for patients of ages > 2 and younger than 19 years with a BMI *>* 95th percentile.

**Methods:**

We performed a retrospective chart review of 417 qualifying encounters to assess adherence in the six months preceding the initiative. We measured adherence as a proportion of eligible patients who had (1) obesity on the problem list; (2) laboratory work offered; (3) counseling provided; (4) early follow-up recommended; (5) referral to a weight management program. In 2018, a multidisciplinary QI team conducted plan-do-study-act cycles to educate providers on the AAP recommendations and improve obesity-related care systems. The initiative lasted 18 months.

**Results:**

During the initiative, we tracked 885 patient encounters via chart review. We witnessed continued improvement in 4 out of 5 measures. For early follow-up offered, we saw improvement after PDSA 1, followed by a decline after PDSA 3. Providers ordered laboratory tests in only 13% of encounters for eligible children ages younger than 6 years versus 45% for ages older than 6 years, an age-dependent disparity that persisted despite the QI initiative.

**Conclusion:**

Our pediatric practice sustained improvement in adherence to AAP recommendations. There is a need to assess the reasons behind the care disparity based on patient ages.

## INTRODUCTION

Obesity is defined as an age- and sex-specific body mass index (BMI) at or exceeding the 95th percentile and has significant comorbidities. Between 2015 and 2016, obesity affected about 13.7 million children and adolescents in the United States.^1^

The standard of care for all patients recommended by the American Academy of Pediatrics (AAP) includes measuring BMI and screening for obesity-related comorbidities. If a child screens positive for obesity, the 2007 Expert Committee Recommendations state that providers should obtain fasting glucose and fasting lipid profile along with alanine aminotransferase and aspartate aminotransferase for children ages 2–18 years.^2^ Additionally, the Endocrine Society recommends using hemoglobin A1C to screen for diabetes. In addition to the appropriate medical screening, AAP also recommends providing positive reinforcement for healthy behaviors in a staged approach with greater intensity as needed.^2^ The United States Preventive Services Task Force, based on grade B evidence, recommends screening for children six years and older and offering at least 26 contact hours/y of intensive behavioral treatment.^3^ Despite this position, screening for obesity and recommended management at well-child checks by primary care providers is not done consistently.^4–7^ In 2013, only one-fourth of a thousand graduating pediatric residents felt that their pediatric obesity management was effective.^8^ However, a recent survey reported that compared with 2006, in 2017, pediatricians were more likely to discuss family behaviors related to screen time, sugar-sweetened beverages, and eating meals together, and were more likely to document BMI.^9^ We may partially attribute this improvement to provider educational initiatives and electronic medical records (EMR)-based interventions.^10–13^ Engagement with positive behavioral changes is challenging for both the patient and family, and an assessment of motivation by the clinician is often time-intensive. Lack of training in obesity management, as well as inadequate reimbursement for obesity-related services including nutritional counseling, is often cited as a barrier to delivering appropriate care.^14,15^

**Fig. 1.**
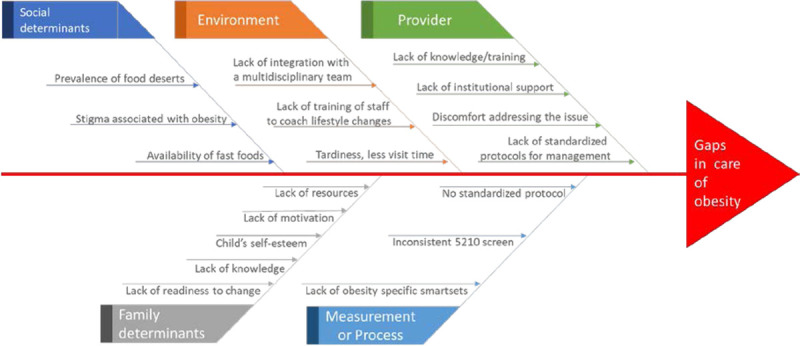
Fishbone diagram. Reasons for suboptimal care process.

We performed this improvement project at the General Ambulatory Pediatric (GAP) clinic at Children’s Hospital at Dartmouth (CHaD). Before outset, our clinic lacked a standardized system of screening for and managing obesity. From our discussions with the providers, we identified several challenges to adhering to recommendations (Fig. 1), and there was variation in care provided to patients with obesity. Examination of a 6-month sample revealed that only 47.5% of patients with obesity had obesity listed on their problem list, 22.3% had referrals made to a weight management program, and 21.8% had screening laboratory tests done. Seeing an opportunity for improvement, we formed a team to carry out a quality improvement (QI) initiative to address this care gap. This project aimed to improve provider adherence to the AAP recommendations for care of patients with obesity by making systematic changes in our practice for all patients ages >2 and <19 with a BMI percentile >95th seen for a preventive visit. Specifically, we aimed to improve the rate of laboratory screening, obesity documentation on the problem list, referral to weight management programs, provision of lifestyle counseling, and weight-specific follow-up visits.

## METHODS

### Context

The GAP clinic is a rural academic tertiary care center serving approximately 18,000 children in the community. Physicians, nurse practitioners, and resident physicians provide patient care. Our setting is the only center in New Hampshire with an intensive weight management center on-site for pediatric patients. This program brings together experts trained in the management of obesity to address obesity to promote healthy lifestyles.

In January 2018, we established a multidisciplinary improvement team consisting of key stakeholders, including pediatric primary care physicians, a registered nurse, licensed nursing assistant, QI analyst, EMR analyst, pediatric clinic leadership, and a physician certified in obesity medicine. Our team reviewed current obesity care practice, published recommendations, identified gaps between practice and evidence through various meetings with our providers, and identified improvement areas. Based on our discussions with the providers, we created a fishbone diagram (Fig. 1) to evaluate barriers to adherence to AAP recommendations. We sought to address several of the provider, environmental, and process-related barriers through this initiative. We used the Institute for Healthcare Improvement’s Model for Improvement as the framework for this effort. This model included developing an aim statement with key drivers, executing plan-do-study-act cycles (PDSAs), and system changes while tracking improvement with control charts. We conducted a retrospective review to obtain baseline adherence to recommendations followed by prospective tracking of our measures to assess our PDSA cycles’ impact.

### Intervention

Table 1 outlines the details of the PDSA cycles conducted during this initiative. These included (1) educational sessions; (2) provision of practice support tools; (3) motivational interview training; (4) engagement of providers and staff for creating a culture of change; and (5) creating a Best Practice Advisory (BPA) in the EMR. Based on our Fishbone diagram, we selected providing education as the first PDSA to address providers’ lack of knowledge. We chose all subsequent PDSAs to address the provider-identified barriers when we fed-back data to them. We have listed these in the “Act” section of the PDSA in Table 1.

**Table 1.** Details of 5 PDSA Cycles Conducted by QI Team

	PDSA Cycle 1 January–April 2018		
Plan	Do	Study	Act
Barriers: Lack of knowledge/training, discomfort addressing obesity as identified by fishbone diagram Goals: Improved knowledge of current recommendations for care demonstrated by improvement in our study measures that in statistical process control	• Quarterly faculty meetings to provide educational material on pediatric-specific obesity • Introducing obesity care in pediatrics in resident continuity care curriculum • 2 resident conferences to provide education on current evidence and gaps in care • Provide access to a detailed overview of the obesity issue through Institute of Healthy Childhood Weight modules that included (1) a detailed overview of the obesity issue; (2) guidance for using an algorithm resource in primary care; and (3) managing and treating obesity comorbidities	• Laboratory testing rates from 21.8% to 25%. • Documentation of obesity on the problem list increased from 47.5% to 52.7% • Referral rates dropped from 22.3% to 18.3% • Documentation of counseling dropped from 64.5% to 58.1% • Follow-up for an obesity-specific visit increased from 10.8% to 16.9%	• When data were shared back with providers they expressed that competing priorities often led to them not follow recommendations • Some identified forgetfulness about what laboratories or historical features that need to be screened for • Others reported that they completed the required screening and counseling but failed to document it
	PDSA Cycle 2 April–August 2018		
Plan	Do	Study	Act
Barriers: Lack of prompts in the EMR to serve as reminders and lack of algorithms for screening readily available in clinics Goals: Improved knowledge of current recommendations for care demonstrated by improvement in our study measures that are in statistical process control	• Provide practice support tools like the AAP algorithm for screening and management of obesity readily available in clinics and provider workspaces • Activity and nutrition materials were to be made available in clinics and workspaces. • Creating an EMR smart-list to support providers in following recommended guidelines	• Laboratory testing rates from 25% to 34.4% • Documentation of obesity on the problem list increased from 52.7% to 55.7% • Referral rates increased from 18.3% to 23.4% • Documentation of counseling increased from 58.1% to 63.6% • Follow-up for an obesity-specific visit increased from 16.9% to 20.6%	• EMR smart-list required the providers to remember to request the smart-list to populate, which was an additional step in the care process. Competing priorities and time constraints were identified as an ongoing barrier • They identified continued discomfort by some to address obesity as an issue given the sensitive nature of the problem
	PDSA Cycle 3 August–December 2018		
Plan	Do	Study	Act
Barriers: Time constraints and competing priorities leading to not using the smart-list when indicated. Continued discomfort with addressing obesity Goals: Improvement in the documentation of counseling provided	• Conduct motivational interviewing workshops for the staff • To make online resources for motivational interviewing training available to staff from the Institute of Healthy Childhood Weight website.	Rates of counseling increased from 63.6% to 65.9% during this PDSA cycle • Laboratory testing rates from 34.4% to 37.7% • Documentation of obesity on the problem list changed from 55.7% to 54.7% • Referral rates increased from 23.4% to 25.7% follow-up for an obesity-specific visit dropped from 20.6% to 12.2%	Providers found the MI training to be helpful They suggested that the drop in early follow-up may have been due to an increase in referrals to a weight management program There continued to be missed opportunities that providers attributed mostly to time constraints
	PDSA Cycle 4 December 2018–April 2020		
Plan	Do	Study	Act
Barriers: Time constraints and missed opportunities to address obesity care Goals: Creating a change culture that would motivate providers, nurses, and flow staff to introduce healthy lifestyle habits. Effectiveness would be demonstrated by improvement in our study measures that are in statistical process control	• Engage the providers and staff and select practice champions to help with improvement efforts. • Carry out improvement huddles to anticipate problems, review performance, and support a culture of improvement. • Brainstorm ideas with all staff for improvement. These resulted in having a theme of the month for healthy eating. Based on those themes, recipes will be sampled for staff before grand rounds These same recipes would be placed in a visible spot in all clinic rooms. Rooming staff will provide these to families while they waited along with some tips/tricks for healthy lifestyles. This would serve as a conversation opener between families and providers about healthy lifestyle	• Laboratory testing rates from 37.7% to 42.9% • Documentation of obesity on the problem list increased from 54.7% to 66.9% • Referral rates increased from 23.4% to 25.7% • Rates of counseling increased from 65.9% to 75.4% • Follow-up for an obesity-specific visit increased from 12.2% to 15.4%	Overall this intervention seemed to have all providers and staff excited to have some actionable items available in the clinic room to provide to families There continued to be room for improvement with regard to consistency in following recommendations
	PDSA Cycle 5 December 2018–April 2020		
Plan	Do	Study	Act
Barriers: Lack of usage of smart-list in the EMR as it required provider to request it in the note. Often times providers completed notes after the encounter which did not serve the purpose of this acting as a prompt for provider Goals: Improvement in our study measures that are in statistical process control	• Implement a BPA for patients with BMI% *>*95th that would be a hard-stop to proceed with the visit (Fig. 3). The BPA window remains open until the provider chooses “open smart-set” or “address later” to allow for clinical judgement in addressing obesity	• Laboratory testing rates from 42.9% to 44% • Documentation of obesity on problem list decreased from 66.9% to 61% • Referral rates decreased from 33.1% to 30% • Counseling rates decreased from 75.4% to 71% • Follow-up for an obesity-specific visit increased from 15.4% to 18%	Hold further PDSA cycles allowing for the current system to be more consistently adopted

### Study of the Intervention

Our EMR analyst reported monthly data. A physician on the team verified this data by chart review. We then fed-back de-identified aggregate data to the practice and individual providers in a monthly report.

### Measures

Outcomes of interest included bimonthly: (1) percentage of patients who had obesity on the problem list; (2) percentage of qualifying patients who had any recommended laboratory tests done in the last 2 years or recommended at the current visit; (3) percentage of patients who were offered to return early for a follow-up; (4) percentage of patients who were referred either to an intensive weight management program or nutrition; and (5) percentage of patients who were provided with lifestyle counseling. The numerator and denominator both were unique preventive care encounters. If a patient had more than 1 eligible encounter during the study period, it was considered a new encounter. This offered another opportunity for the provider to suggest recommended care. We chose obesity on the problem list as a proxy for the identification and classification of BMI. All other measures are consistent with the AAP recommendations for identifying, screening, and managing pediatric obesity in primary care, aligning with this project’s aim.^2^ We considered the provision of lifestyle counseling done if any one of the counseling elements recommended by the AAP were addressed and documented in the assessment and plan. These include limiting sugar-sweetened beverages, encouraging consumption of fruits and vegetables, limit eating out, portion control, limiting screen time, and increasing activity, among others.^2^ The 5,210 model was frequently used, which is a nationally recognized obesity prevention program.^16^ This program encourages children to consume at least 5 servings of fruits/vegetables in a day, limit screen time to less than 2 hours a day, increase physical activity to at least an hour a day, and avoid sugar-sweetened beverages. Table 2 outlines these measures and their operational definitions. The 5 measures were classified as process measures and were derived from prior studies showing that changes to them led to an eventual decrease in patients’ BMI.^2,3^ Although mere adherence to these measures without follow-up is not enough, these are evidence-based first steps under the responsibility of the provider that lead to improved care for obesity.^3,17–19^

**Table 2.** Operational Definitions of Process Measures

Measure	Operational Definition
Obesity on problem list	Percent of eligible encounters that had obesity documented in the problem list or any other weight-related concerns in the problem list like overweight or high BMI percentile.
Referral	Percent of eligible encounters that were offered a referral to pediatric lipid and weight management program, nutrition or a community-based pilot program either at this visit or these orders were placed ever in the past. This was tracked by noting documentation of “referral offered” in the progress note or actual referral order noted in EHR.
Laboratory tests	Percent of eligible encounters that had any of the screening labs offered at this visit or done in the last 2 y. These include: (1) lipid profile; (2) ALT/AST; and (3) A1C or fasting blood glucose
Counseling	Percent eligible patients who had any documentation of counseling around healthy lifestyle/weight done at current visit. Any documentation of discussion endorsing healthy diet and activity will count
Recommended early follow-up	Percent eligible encounters where a follow-up was offered to discuss BMI percentile and obesity

Denominator for all measures is patient encounters between ages >2 and younger than 19 years seen for preventive visit during the study period.

A1C, hemoglobin A1C; ALT, alanine aminotransferase; AST, aspartate aminotransferase; EHR, electronic health record.

### Evaluation Methods and Results Analysis

Monthly reports of all patients ages >2 and younger than 19 years with BMI >95th and a preventive visit at GAP were queried, filtered, and plotted on p-charts.^20^ We used established rules for differentiating special versus common cause variation for these charts.^20^ We conducted SPC (p-chart) analyses using the QI Macros package implemented in Microsoft Excel 2016 software (KnowWare International Inc, Denver, Colo.). P-chart centerlines and control limits were recalculated when ≥7 consecutive data points were above/below the centerline average, representing a special cause signal shift. We used inferential statistics z-test scores for differences between proportions (alpha < 0.05) and mean differences with 95% confidence intervals for baseline and intervention comparative analyses.

We also performed an age-based subanalysis using the following parameters from the WHO: (1) Group A: 2 to < years; (2) Group B: 6 to <13 years; and (3) Group C: 13 to <19 years to assess how our practice changed depending on patient age. Our team collected baseline data for the year 2017 via chart review of a total of 417 qualifying encounters. We initiated our project in January 2018 with the implementation of PDSA cycle 1 (Table 1).

### Ethical Concerns

The Dartmouth Committee for the Protection of Human Subjects deemed the project as QI and, therefore, was exempt from review.

## RESULTS

**Fig. 2.**
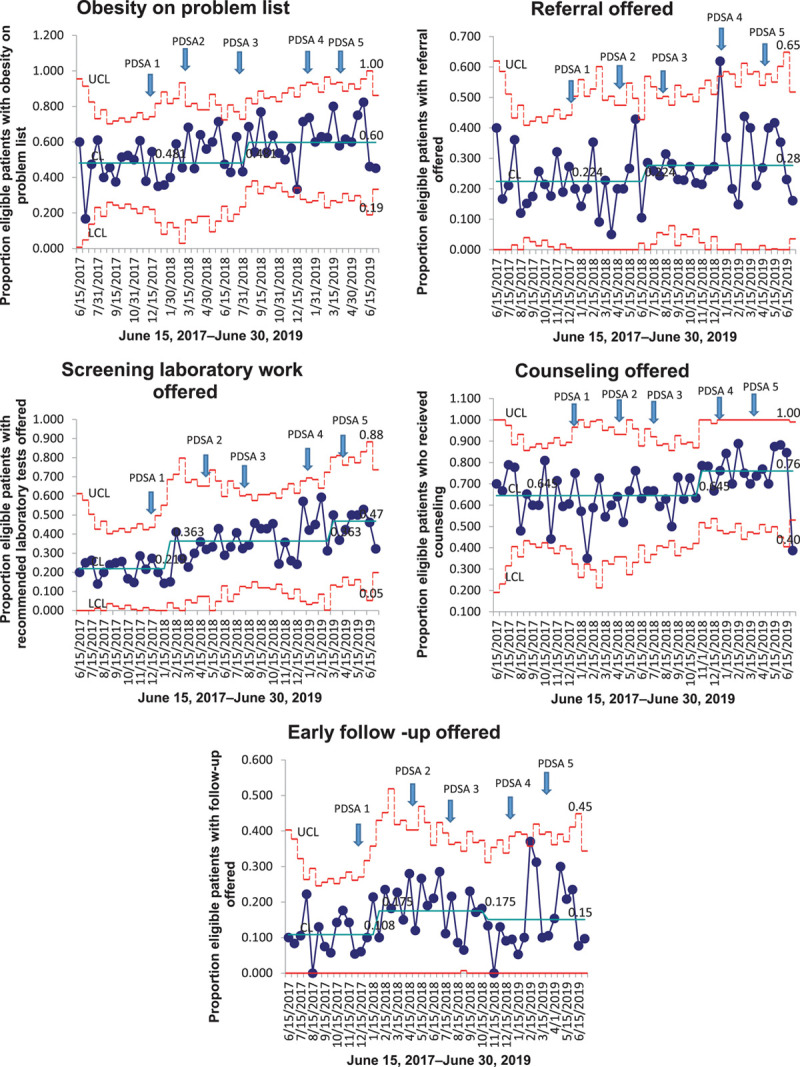
P-charts for all 5 measures tracked showing the proportion of eligible patient encounters that satisfied our process measures.

Throughout this initiative, 885 eligible patient encounters took place. We tracked our improvement measures prospectively. We observed p-chart special cause signal shifts for all five measures.^20^ We witnessed continued improvement in 4 out of 5 measures. For early follow-up offered, we saw improvement after PDSA 1, followed by a decline after PDSA 3. Figure 2 displays the p-charts showing prospectively tracked data.

When comparing baseline versus intervention period rates, we noted statistically significant improvement in 3 out of 5 measures. The percentage of encounters where obesity was listed in the problem list significantly increased from 47.5% at baseline to 57.5% during the initiative (*P* < 0.01). Laboratory screening rate significantly increased from 21.8% to 37.9% (*P* < 0.01). Recommended early follow-up rates increased from 10.8% to 16.4% (*P* < 0.01). In the encounters tracked during our initiative, 21.4% of children were in the 2 to <6 years age, 42.9% in the 6 to <12 years age, and 35.6% in the 13 to <19 years. The average BMI percentiles for the 3 groups were 98, 97.8, and 97.7, respectively. Table 3 outlines age-based adherence to recommendations at baseline compared to the intervention period. Adherence remained low for 2–6 years old throughout the study period (Table 3).

**Table 3.** Percentage Compliance by Age at Baseline and Intervention Period

	Age 2 to younger than 6 years					Age 6 to younger than 13 years					Age 13 to younger than 19 years				
	Baseline		Intervention			Baseline		Intervention			Baseline		Intervention		
	n	%	n	%	*P*	n	%	n	%	*P*	n	%	n	%	*P*
Obesity on problem list	17	21.0	76	40	0.0025	95	50.8	220	57.9	0.1096	85	57.8	215	68.0	0.0324
Recommended early follow-up	19	23.5	16	8.4	0.0008	28	15.0	62	16.3	0.6892	61	41.5	66	20.9	0.0000
Referral	5	6.2	14	7.4	0.7263	38	20.3	108	28.4	0.0385	16	10.9	110	34.8	0.0000
Laboratory tests	5	6.2	24	12.6	0.1188	43	23.0	151	39.7	0.0001	50	34.0	160	50.6	0.0008
Counseling	45	55.6	122	64.2	0.1835	118	63.1	256	68.2	0.2263	106	72.1	222	70.3	0.6892

**Fig. 3.**
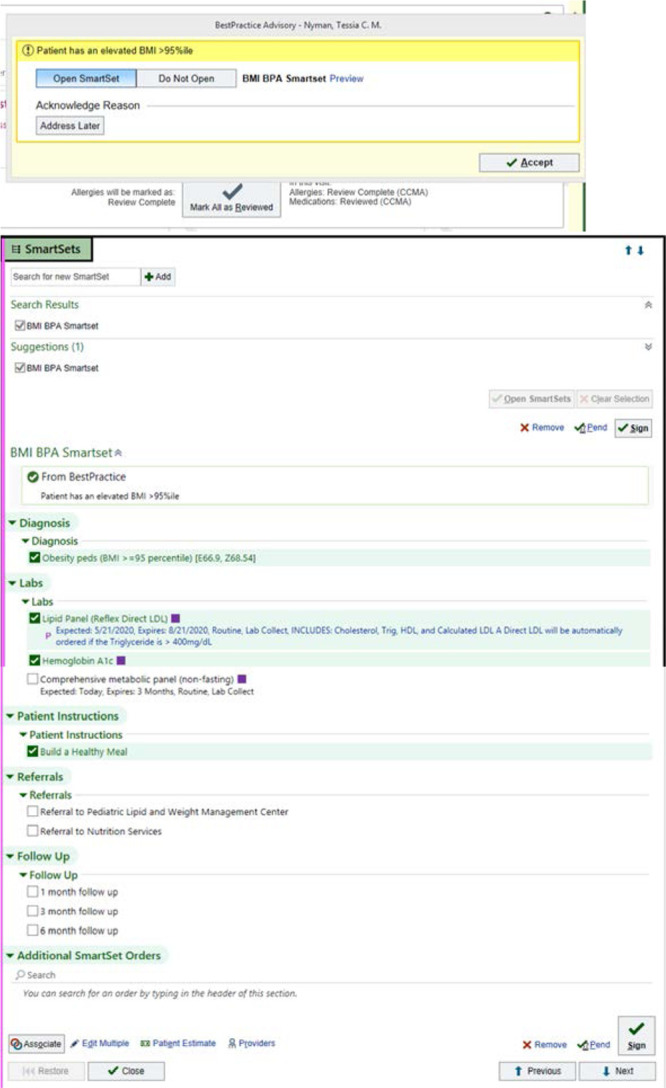
BPA linked to a smart-set.

We have listed key improvement areas and specific interventions for the initiative in Table 1. Key interventions that were tested and implemented included providing education to all staff, implementing changes to EMR to improve documentation, motivational interview training, involving the staff in creating handouts and recipes for patients, and a BPA in the EMR (Fig. 3).

## DISCUSSION

We describe a QI initiative to standardize care provided to pediatric patients with obesity by promoting adherence to the AAP recommendations. This initiative led to an improvement in all 5 process measures. Our team conducted 5 PDSA cycles and implemented several changes during this initiative. We met with the providers to identify reasons for change or lack of it to determine why we saw specific changes. Based on these discussions, the core QI team suggested the next PDSA cycle to the group. Changes implemented during PDSA 1 primarily focused on educational efforts directed towards healthcare professionals. These changes resulted in an improvement in screening laboratory work being suggested, and offering “early follow-up.” Several previous studies have confirmed that pediatric health professionals have often failed to diagnose childhood obesity or provide lifestyle counseling.^4,6,21,22^ We attribute this partially to a lack of obesity-related education provided to trainees, leading to providers feeling ill-prepared to tackle the issue.^23,24^ PDSA 2 focused on making tools readily available for providers to have easy access to recommendations when providing patient care. With this intervention, we were able to observe improvement in the percentage of eligible patients referred to specialty care. PDSA 3 focused on motivational interviewing techniques to further empower providers in delivering their message effectively to patients. With this, lifestyle counseling rates improved but surprisingly, offering an early follow-up appointment decreased but remained above the preintervention rates.

PDSA 4, focused on the culture of clinical practice change, resulted in another special cause signal in offering screening laboratory work. This included involving the entire clinic staff in the QI effort by creating obesity champions within the practice. These individuals were nurses, medical assistants, and physicians with a particular interest in obesity. Studies show that primary care practices are most successful at adopting QI culture when they can identify a change champion, described as a leader who can enable change and be willing to embrace and model innovative practices.^25^ As our practice brainstormed ideas during the PDSAs described in Table 1, the clinic identified practice champions. For instance, the registered dietician on the QI team helped create healthy recipes and meal preparation demonstrations. They implemented several changes in this phase: (1) conducted meal preparation demonstrations; (2) created monthly themes for healthful lifestyle; (3) trained the rooming staff to introduce recipes to families and healthy plate information; (4) supported staff and medical assistants in further training in obesity care by attending beneficial conferences. Notably, many of these interventions were led and driven by the clinic, which created a culture change. Active participation by end-users in the design and implementation of improvement efforts increases the team buy-in for the change because they attribute a higher value to the improvement.^26,27^ We also offered Maintenance of Certification points to clinicians participating in the initiative as an added incentive.

In the 3 months following BPA introduction, we did not observe a special cause signal in any measures, although prior improvement gains were sustained. We noticed alarm fatigue when the BPA was first introduced. Providers reported that the alert fired every time they accessed a chart. We corrected this by making an EMR change to ensure that the BPA fired only for the encounter provider and only once in 24 hours. We also ensured that the BPA did not fire for acute visits. Change fatigue is well documented in improvement efforts and could have contributed to lack of improvement after this intervention.^28^ We chose PDSA 5 based on the success of BPAs in previous QI efforts for other disease processes and feedback from our providers at the end of prior PDSAs.^29–31^ The BPA was quickly taken up by other CHaD primary care offices outside of our clinic, including family medicine and CHaD pediatrics in other cities. In sum, results from our QI initiative suggest that system-wide changes which encourage adopting standardized practice approaches to obesity management in primary care can improve adherence to expert recommendations.

Our study had numerous strengths, including data reliability verified by chart review. We had a highly engaged multidisciplinary team, as described above. We were able to track our measures prospectively over 18 months. We chose an issue relevant to primary care pediatrics in a setting with sufficient resources to address the problem when identified. We attained a significant improvement in our measures that will hopefully extend beyond the current initiative as new systems are now in place to support providers and engage staff and patients. We learned that BPAs served only as reminders and that improved practice requires a culture of improvement with involvement and engagement at all levels, including the macro, meso, and microsystem.

Another important observation was that adherence rates for all five measures remained low in the 2- to 5-year age group despite our QI efforts. Previous studies have shown that preschool-aged children have a drastic change in their diets, switching to more nutrient-poor and energy-rich foods.^32^ Evidence also indicates that most excess weight before puberty is gained before 5 years of age and is predictive of weight at nine years.^33^ Our study furthers this work by identifying a potential age bias in which obesity is left underrecognized and undertreated in the youngest and most vulnerable patient population, a significant opportunity for future improvement. Elimination of healthcare disparities is a key focus of many national agencies, including the Centers for Disease Control. Our preintervention data analysis identified an age-based difference in pediatric obesity management despite consistent recommendations for ages 2–18 years.^2^ Although our initiative did not focus on identifying the reasons for this disparity, we believe it is essential to document and report these differences. Adherence remained low in this age group, but we noted significant improvement in obesity documentation on the problem list and referrals made to a weight management program. Other potential contributors to obesity care disparities, including socioeconomic status, gender, parental education level, were not tracked and will be part of our group’s future efforts.

### Limitations

Our improvement initiative had some limitations. Although the inclusion of balancing measures is optimal for QI efforts, we were unable to identify a meaningful balancing measure. We think it is unlikely that our patients’ or other patients’ care was compromised due to our efforts as we were only tracking well visit encounters. Another limitation is that we did not measure the knowledge gap preintervention and postintervention through a survey. However, we did meet with the trainees and providers before the intervention to create a fishbone diagram, through which many identified that they were not aware of the AAP recommendations. We addressed this through PDSA 1. Last, we included obesity on the problem list as a proxy for identifying and screening for obesity, which may not be an accurate proxy; however, there is existing evidence that adding obesity to the problem list improves providers’ rates of addressing obesity.^34^ Accordingly, it is critical to document obesity in the patient’s EMR so that the information is used to improve care and health communication and, in turn, the patient’s prognosis. As shown in Table 2, we also included patients who had “overweight” in their problem list as having “obesity on the problem list.” We did this as some pediatricians in our practice reported that they avoided using “obesity” and instead used “high BMI” or “overweight” due to the stigma associated with the word obesity. However, we only included patient encounters with BMI >95%, so this did not result in the inclusion of overweight patients.

## CONCLUSIONS

In summary, our pediatric practice demonstrated improved adherence to AAP recommendations for screening and management of pediatric obesity through systematic changes. We speculate that key elements leading to this project’s success can be linked to effective local site champions who encouraged and modeled change and improvement-minded institutional leaders who encouraged and supported change, particularly in the EMR. One of our key next steps will include investigation into the reasons for the age-related care disparity.
